# On the Entropy of a One-Dimensional Gas with and without Mixing Using Sinai Billiard

**DOI:** 10.3390/e23091188

**Published:** 2021-09-09

**Authors:** Alexander Sobol, Peter Güntert, Roland Riek

**Affiliations:** Laboratory of Physical Chemistry, ETH Zurich, 8093 Zurich, Switzerland; kristina.comiotto@phys.chem.ethz.ch (A.S.); peter.guentert@phys.chem.ethz.ch (P.G.)

**Keywords:** entropy, Gibbs factor, ideal gas, Sinai billiard, Stirling formula, causality one dimensional gas

## Abstract

A one-dimensional gas comprising *N* point particles undergoing elastic collisions within a finite space described by a Sinai billiard generating identical dynamical trajectories are calculated and analyzed with regard to strict extensivity of the entropy definitions of Boltzmann–Gibbs. Due to the collisions, trajectories of gas particles are strongly correlated and exhibit both chaotic and periodic properties. Probability distributions for the position of each particle in the one-dimensional gas can be obtained analytically, elucidating that the entropy in this special case is extensive at any given number *N*. Furthermore, the entropy obtained can be interpreted as a measure of the extent of interactions between molecules. The results obtained for the non-mixable one-dimensional system are generalized to mixable one- and two-dimensional systems, the latter by a simple example only providing similar findings.

## 1. Introduction

The second law of thermodynamics states that the entropy *S* of an isolated system increases monotonically towards its thermodynamic equilibrium value [1,2,3,4,5]. Following the systematic formulation of statistical mechanics by Gibbs and Boltzmann, the entropy $S^{G}$ is a measure of the number of accessible micro-states of the system of interest in its given macro state. For a micro-canonical ensemble the entropy is given by

(1) $$S^{G}=k_{B}\ln\Omega$$

with $k_{B}$ the Boltzmann constant and Ω the number of accessible micro-states describing the macro-state [2,3,4,5,6,7]. In order to describe the entropy of a micro-canonical isolated system in textbooks simple probability theories are used with imaginary boxes as well as the use of the first order Stirling approximation, lnN!≈NlnN−N with an error proportional to lnN, which poses issues in the thermodynamic limit (N→∞) [8,9,10,11], unless the concept of an entropy density [12], or even redefining the microscopic origin on entropy [13,14] is introduced. Instead of using these somewhat ad hoc models to describe a micro-canonical ideal gas, we study here a one-dimensional gas comprising *N* point molecules that undergo elastic collisions within a finite space because it can be analytically calculated using the Sinai billiard approach. The one-dimensional gas is special in the sense that there is no mixing (collisions prevent particles from “overtaking” each other), the particles are distinguishable (particles numbered initially according to their spatial position always remain in that order), and it fulfills the ideal gas equation despite of the strong interaction by the elastic collisions. As we shall demonstrate, the analytical calculation of the particle position probability distribution for the one-dimensional gas using Sinai billiard yields $N^{N}$ as the correct Gibbs factor and not the heuristically introduced N!. This derivation further illustrates that the entropy is dictated by the interaction between particles. After a quantitative analysis of the one-dimensional gas in Section 2.1 and Section 2.2 its analytical analog is derived using the Sinai billiard approach in Section 2.3. In Section 2.4, a recoloring concept is introduced to derive the entropy, followed by a two-dimensional extension of this concept via an illustration by examples (Section 2.5), and by a revisitation of the Gibbs paradox in Section 2.6. The results obtained are discussed thereafter (Section 3).

## 2. Theory

### 2.1. Standard Approach to Calculate the Configurational Entropy of a One-Dimensional Ideal Gas

Let us consider a diluted, ideal gas in a (one-dimensional) box with *M* (imaginary) sites and *N* indistinguishable particles of point-like character and equal mass *m* (with M≫N because the gas is diluted). For simplicity, each site may be occupied with more than one particle, which is possible since the particles are point-like. Following Gibbs (after resolving the Gibbs paradox) the number of micro-states possible is [1,2]

(2) $$\Omega =\frac{M^{N}}{N!}$$

yielding

(3) $$S^{G}=k_{B}\ln\frac{M^{N}}{N!}$$

The number of micro-states is divided by N! because the particles are indistinguishable. Furthermore, it is noted that the configurational space sites of which there are *M* are not further defined here.

Using only the first term (i.e., the first order approximation) of the Stirling formula, i.e., lnN!≈(NlnN−N), which is usually used in standard textbooks on statistical thermodynamics, the following description of the Boltzmann entropy is obtained

(4) $$S^{G}=k_{B}N\ln M-k_{B}\ln N!\approx k_{B}N\ln M-k_{B}\left(N\ln N-N\right)=k_{B}N\left(\ln\frac{M}{N}+1\right)$$

After applying (and only after applying) the first term in the Stirling formula (shown in brackets above) the entropy is of extensive nature [8], provided that the number of sites, *M*, is chosen proportional to the system size, i.e., the number of particles, *N*.

Similarly, a model of a gas that does not allow two particles to occupy the same site yields $\Omega =\frac{M!}{(M-N)!N!}$ and concomitantly

(5) $$S^{G}=k_{B}\ln\frac{M!}{(M-N)!N!}\approx k_{B}N\left(\ln\frac{M}{N}+1-\frac{N}{M}\right)$$

Again, the first order approximation of Stirling’s formula, and ln(1+x)≈x for |x|≪1 were used. Since M≫N, the two models are essentially equivalent. It is evident, that these two presented approaches to calculate the configurational entropy of an ideal gas are equivalent.

### 2.2. A Quantitative and Analytical Description of the Entropy of a One-Dimensional Gas with Particles Having a Constant Absolute Velocity

A one-dimensional gas is described here as *N* single atom entities of point-like character of mass *m* located within a finite space with length *L* (in the following figures L=1). A particle has a position coordinate x∈[0,L] and velocity *v*, which is related to the temperature *T* of the system by equipartition of the kinetic energy, i.e., $\frac{1}{2}k_{B}T=\frac{1}{2}mv^{2}$ and thus $v=\pm \sqrt{\frac{k_{B}T}{m}}$. That is, if all particles have the same absolute velocity. If a particle collides, the collision is of elastic nature, i.e., v→−v at the collision; this includes also collisions with the wall of the finite space.

**Figure 1:**
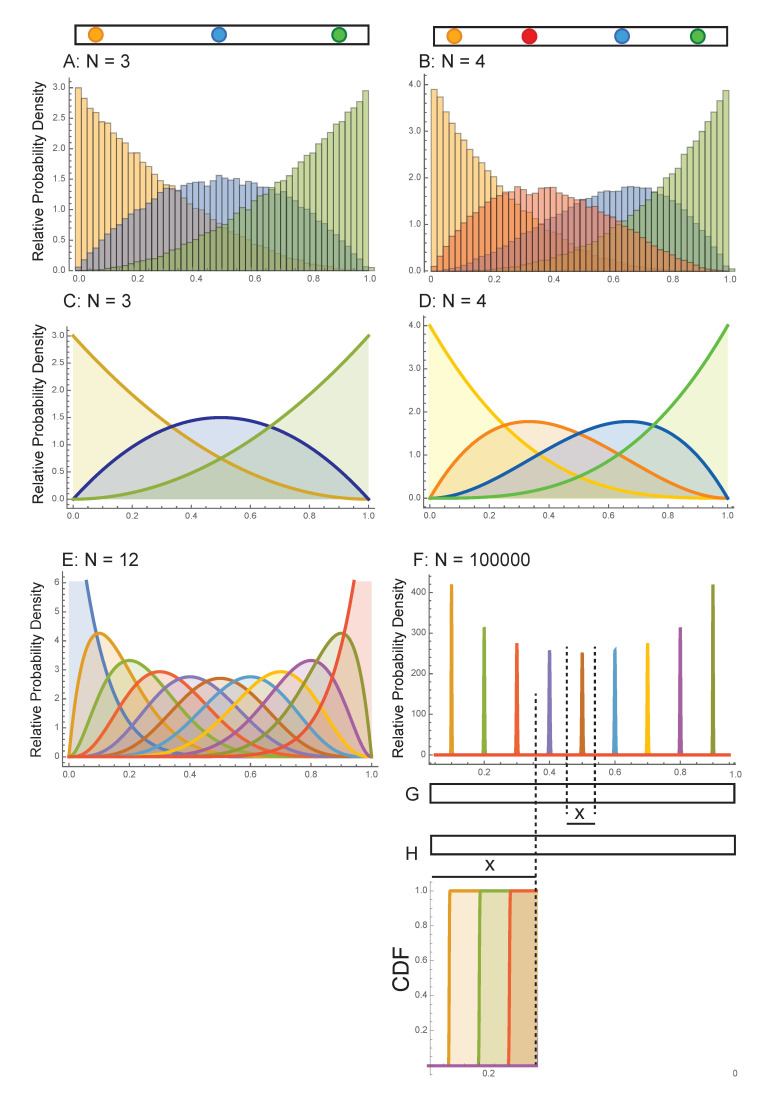
Probability density distributions for particle positions in a one-dimensional gas with elastic collisions: (**A**) simulated for a three-particle system, (**B**) simulated for a four-particle system, (**C**) analytical probability densities given by Equation (6) for a three-particle system, (**D**) four-particle system, (**E**) 12-particle system, and (**F**) 100,000-particle system obtained using the so-called Sinai billiard concept. A cartoon for the two systems at the top shows the point particles as spheres color-coded like their corresponding probability distributions in the graphs. In (**C**), the probability density for the yellow particle is $f\left(x\right)=3{\left(1-x\right)}^{2}$, for the blue particle f(x)=6x(1−x), and for the green particle $f\left(x\right)=3x^{2}$. In (**F**), the probability density is plotted only for every 10,000th particle for clarity. In (**G**), a cartoon indicates the entropy calculation within the virtual small space around position *x* in the middle of the large box with length L=1. In (**H**), another virtual system located in the region [0,x] is indicated. The cumulative probability density functions of the 10,000th, 20,000th, and 30,000th particle within a 100,000 particle system (shown in (**F**)) are plotted below.

For this model, the ideal gas equation, $pV=Nk_{B}T$ with pressure *p* and volume *V*, is fulfilled because $pV=p\;L\;A=\frac{\langle F\rangle }{A}L\;A=\langle F\rangle L$ with *A* the area of the wall perpendicular to the axis of the one-dimensional system and the time-averaged force on the wall, $\langle F\rangle =\frac{1}{\tau }\int_{0}^{\tau }F\left(t\right)dt=\frac{1}{\tau }\int_{0}^{\tau }m\frac{dv}{dt}dt=\frac{1}{\tau }n_{\mathrm{coll}}m\;2v$. The number $n_{\mathrm{coll}}$ of collisions with the wall during the (long) averaging time period τ can be estimated by considering that the particle closest to the wall has to travel, on average, twice the distance L/N between two collisions: $n_{\mathrm{coll}}=\tau /\left(\frac{2L}{Nv}\right)$. This yields $\langle F\rangle =\frac{N}{L}mv^{2}$ and hence $pV=Nmv^{2}=Nk_{B}T$. In other words, this very simple one-dimensional model with constant absolute velocities can be considered a one-dimensional ideal gas. Furthermore, the restriction to a single absolute velocity thereby enables an analytical solution of the entropy as we shall see. However, first a simulation of a typical example of a three-particle system is calculated (see Material and Methods for details). For the position of each particle, the probability density distributions shown in Figure 1A are obtained. The yellow particle is predominantly located in the left part of the space while it may still occupy positions in the entire box. Correspondingly, the blue particle is rather in the middle and the green particle on the right side of the one-dimensional box. In the case of four particles shown in Figure 1B, the location distributions change slightly to accommodate the additional particle and collisions therein. Going from a three- to a four-particle system, the individual distributions get sharper along the space coordinate (see also below).

**Figure 2:**
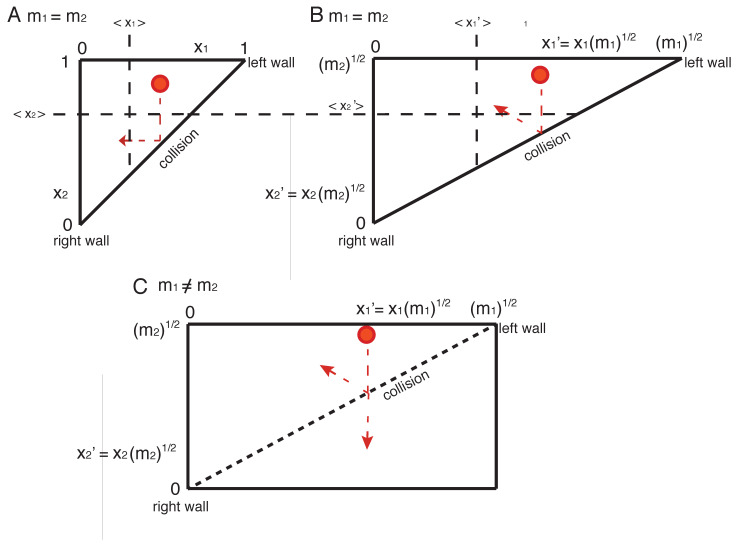
Sinai Billiard for two-particle systems in a one-dimensional setting having (**A**) equal mass, (**B**) unequal mass with $m_{1}>m_{2}$, and (**C**) in the presence of mixing (i.e., particles passing each other instead of colliding). According to Sinai, the motion of a single point particle (exemplified in red) in the two-dimensional triangle is equivalent to that of two point particles in a one-dimensional box. (**B**) To accommodate different masses of the two particles in the one-dimensional box, the triangles side length must be multiplied by the unitless $\sqrt{m_{1}}$ and $\sqrt{m_{2}}$ according to the transformation with $x_{1}^{\prime }=\sqrt{m_{1}}\;x_{1}$ and $x_{2}^{\prime }=\sqrt{m_{2}}\;x_{2}$. In (**C**) The Sinai Billiard for two particles with unequal mass that can mix by tunneling is represented by a single particle in a triangle, that stays within a triangle representing a collision or that leaves the triangle through the collision wall when tunneling. To indicate the pore character of the hypotenuse it is drawn as a dashed line. The average positions $\langle x_{p}\rangle$ and $\langle x_{p}^{\prime }\rangle$ are also indicated.

An analytical description of the probability density can be given by using a generalized Sinai Billiard for which the probability density of a single particle in a *N*-dimensional simplex is equivalent to *N* particles in a one-dimensional box [15,16,17]. The approach is illustrated for a system of two particles with equal masses in Figure 2A.

The single particle in the two-dimensional triangle can be regarded as a one-dimensional system with two particles. The $x_{1}$ axis corresponds to the left wall, the $x_{2}$ axis to the right wall. If the particle comes from the top (along the vertical axis) as shown, it can be considered particle 1, hitting the hypotenuse corresponds to the collision with particle 2, and the following horizontal motion corresponds to particle 2 that eventually hits the right wall. In the example in Figure 1A, the red particle coming from the top, hitting the wall, and going to the left therefore corresponds to a one-dimensional situation where particle 1 comes from the left wall, while particle 2 does not move. After the collision, particle 1 stopped moving, while particle 2 moves towards the right wall. Hence, to study the configurational entropy (or the phase space) of the multi-particle system in a one-dimensional box is equivalent to that of a single particle system in a N-dimensional simplex (with all its properties). From the multi-particle system point of view, according to Sinai [15], the probability density function for the position of particle *p* = 1, …, *N*, numbered from left to right in a box of length L=1, is then given by the beta distribution

(6) fp(x)=1B(p,q)xp−1(1−x)q−1=pNpxp−1(1−x)N−p

for x∈[0,1] and $f_{p}\left(x\right)=0$, otherwise (i.e., outside the box) with q=N+1−p. The beta function is given by $B\left(p,q\right)=\int_{0}^{1}t^{p-1}{\left(1-t\right)}^{q-1}dt=\frac{\Gamma (p)\Gamma (q)}{\Gamma (p+q)}$ in terms of the gamma function $\Gamma \left(z\right)=\int_{0}^{\infty }t^{z-1}e^{-t}dt$. A proof of Equation (6) is given in Appendix A. The probability density $f_{p}\left(x\right)$ is exemplified in Figure 1 for *N* = 3, 4, 12, 100,000.

The mean position of particle number *p* counting from the left is (reintroducing the length *L*)

(7) $$\langle x_{p}\rangle =\frac{pL}{N+1}$$

The mean position can also be obtained studying a single particle in an *N*-dimensional simplex as illustrated for one particle in a two-dimensional space (corresponding to two-particles system in a one-dimensional space) in Figure 2A,B.

From inspection of Figure 1, it is evident that the probability density distributions narrow with increasing number of particles *N*. Analytically this can be described by the standard deviation σ of the beta distribution given by

(8) $$\sigma_{p}=\frac{L}{\left(N+1\right)}\sqrt{\frac{p(N+1-p)}{N+2}}$$

The standard deviation is smallest for particles near the left or right wall, where $\sigma_{p}\approx \frac{L}{N}$ for large *N*, and largest for particles in the middle of the box, where $\sigma_{p}\approx \frac{L}{2\sqrt{N}}$ for large *N*. The standard deviation thus depends on the location of the particle *p*. Actually it is the distance to the wall that matters.

In order to calculate the configurational entropy of this one-dimensional gas system and to study its extensive character three approaches I-III are discussed in the following. In approach I, we continue on the above standard deviation $\sigma_{p}$. We assume each particle to be located within κ times the standard deviation (for instance, κ=3 covers >99% of its location) and the number of micro-states to be proportional to the accessible configuration space (which requests removing the unit of L), i.e.,   

(9) $$\Omega \propto \prod_{p=1}^{N}\kappa \sigma_{p}=\kappa^{N}\prod_{p=1}^{N}\frac{L}{\left(N+1\right)}\sqrt{\frac{p(N+1-p)}{N+2}}=\kappa^{N}{\left(\frac{L}{N+1}\right)}^{N}\frac{N!}{{\left(N+2\right)}^{N/2}}\approx \frac{\kappa^{N}L^{N}N!}{N^{3N/2}}$$

yielding an entropy estimation $S^{G}=k_{B}\ln\Omega$ that is not extensive, i.e., $S^{G}\left(\lambda L,\lambda N\right)\neq \lambda S^{G}\left(L,N\right)$, even if we use the first order Stirling approximation. This is related to the non-local influence of the walls that influence the standard deviation of the particle positions not only in their vicinity, but over the entire system. The corresponding standard deviations of a system scaled by a factor λ in size and number of particles are approximately $\sqrt{\lambda }$ fold larger than for the original system, $\sigma_{\lambda p}\left(\lambda L,\lambda N\right)\approx \sigma_{p}\left(L,N\right)\sqrt{\lambda }$, whereas they should remain constant for extensivity. That is, boundary effects decisively influence the bulk properties of the system. This is not compatible with extensivity of a macroscopic thermodynamic system that can in general only be realized if boundary effects are negligible.

In approach II, we therefore calculate the accessible configuration space of a (sub)system of length l≪L comprising n≪N particles located near the center of the original system (Figure 1G). Such an approach is often done in theoretical calculations to get rid of issues with the wall (i.e., boundary effects). The *n* particles of the subsystem thus correspond to particles $p\approx \frac{N}{2}$ of the original system. Assuming that the particles are located within κ times the standard deviation from their mean position and that the number of micro-states is proportional to the accessible configuration space, this yields the number of micro-states

(10) $$\Omega \propto {\left(\kappa \sigma_{N/2}\right)}^{n}\approx {\left(\frac{\kappa L}{2\sqrt{N}}\right)}^{n}={\left(\frac{\kappa^{2}L^{2}}{4N}\right)}^{n/2}={\left(\frac{\kappa^{2}Ll}{4n}\right)}^{n/2}$$

for the *n*-particle subsystem of length *l* in the center of the *N*-particle system of length *L* (and thus n/l=N/L). The Boltzmann entropy is then given by

(11) $$S^{G}=k_{B}\frac{n}{2}\left(\ln\frac{l}{n}+\mathrm{const}.\right)$$

Note that ρ=n/l is the particle density and thus the term within the logarithm is of intensive nature and the constant factor $\frac{\kappa^{2}L}{4}$ was moved into the const., which is possible since both $\kappa^{2}$ and *L* are within this calculation arbitrary parameters (it is also noted that the term $\kappa^{2}$ results from the selection criterion of κ standard deviations). In the case of calculating entropy differences it cancels out and is thus of no importance.

This yields an entropy description that is of extensive character for any number of particles, *n*, located in the middle of a large box such that boundary effects can be neglected. This is in contrast to the standard entropy of an ideal gas that holds only in the thermodynamic limit with n→∞ (and this only under the assumption that the first order term of the Stirling formula can be used, but see [8,18]). The entropy calculated here is proportional to the number of particles of interest, *n*, and proportional to the logarithm of the particle density. Note that the present derivation does not differentiate between distinguishable and non-distinguishable particles.

Comparing the standard configurational entropy of the ideal gas (Equation (3)) with the here derived one-dimensional analog of Equation (11) reveals that N! is replaced by $N^{N}$ (in order to return to the general formalism, we again write *N* instead of *n*). This difference has major consequences. As described, it makes entropy extensive for any particle number and without assuming the Stirling formula. Furthermore, it gives the space-to-particle number relationship a prominent role. In the case of the one-dimensional gas calculations in Figure 1, it is evident that the particles j≠p influence the position of particle *p* and its location distribution. The more particles there are, the narrower the spatial distribution of particle *p* becomes. In other words, the presence of all the (other) particles *j* restricts the space of any given particle *p* (Equation (9)) and determines the mean location and standard deviation (Equations (7) and (8)) of particle *p* via the density. Accordingly, the entropy is affected by the interactions between particles.

In approach III, we follow Boltzmann’s concept by determining the probability of a system to be in a given macro state [2]. For the one-dimensional cases this means that the probability for a particle to be in the region [0,x] (with 0<x≤L) is calculated by the cumulative distribution function $F_{p}\left(x\right){=}_{0}^{x}f_{p}\left(x^{\prime }\right)dx^{\prime }$ of the beta distribution for each particle *p* with a mean position $\langle x_{p}\rangle <x$. Figure 1H shows the cumulative distribution function of several particles at distributed locations. It is evident that for the particles with mean position $\langle x_{p}\rangle =\frac{pL}{N+1}<x$, the cumulative probability $F_{p}\left(x\right)$ is very close to 1, whereas for all particles with $\langle x_{p}\rangle >x$, this probability is almost 0. Because of the shape of the positional probability distributions, it is assumed that the configuration space for each particle is $\frac{L}{N}$ and that the number of micro-states $M\propto \frac{L}{N}$. This yields $\Omega =M^{N}$ and, without further approximations, a strictly extensive entropy

(12) $$S^{G}=k_{B}N\ln M=k_{B}N\left(\ln\frac{L}{N}+\mathrm{const}.\right).$$

In summary, three approaches are presented to calculate the configurational entropy of the one dimensional gas with constant absolute velocity introduced in the beginning of Section 2.2 quantitatively (Figure 1). All three are analytically solved and valid because usually entropy differences are calculated. Approach I includes the effect of the wall, and as such is not extensive because a merge of two systems with each two walls would yield a reduction of the number of walls by two. Approaches II and III circumvent the issue with the wall and allow the study of entropy extensivity.

### 2.3. Determining the Entropy of a One-Dimensional Ideal Gas by a Recoloring/Renumbering Concept

Again a one-dimensional ideal gas is described as *N* indistinguishable entities of point-like character within a finite space with length *L*. If a designated particle (either numbered or colored as in Supplementary Movie S1) collides with another particle, then at the moment of collision the number/color of the two particles can be exchanged as the two particles are indistinguishable. With this renumbering/recoloring scheme it appears that each particle—in the following called pseudo-particle—moves inside the one-dimensional box at constant velocity without any collisions, but those with the walls covering thereby M/N distinct micro-states with $M=\frac{L}{\Delta x}$ spatially localized micro-states where Δx<<L/N is the spatial extension of a micro-state. The division by *N* is due to the number of collisions (i.e., recoloring events or exchange between two particles) from wall to wall reducing the number of micro-states accordingly. Furthermore, each pseudo-particle behaves on average the same and moves independent of the others. The total number of such micro-states for the entire system of *N* pseudo-particles is then

(13) $$\Omega ={\left(\frac{M}{N}\right)}^{N}={\left(\frac{L}{N\Delta x}\right)}^{N}$$

yielding the entropy

(14) $$S^{G}=k_{B}N\left(\ln\frac{L}{N}+\mathrm{const}.\right)$$

### 2.4. Extensions to Two Dimensions

The situation with the one-dimensional ideal gas is special in the sense that each particle is distinguishable. For instance, particle number 9 from the left always remains number particle 9 because the one-dimensional nature of the system and the collisions make it impossible for particles to pass each other (but see Section 2.6). One may thus argue that the “normalization” $N^{N}$ presented here is valid only for the one-dimensional ideal gas and not for two- and three-dimensional systems. In the following section, the above arguments are extended to the two-dimensional ideal gas (described by *N* indistinguishable point particles within a finite two-dimensional space of size L×L) by an argumentation only using two different approaches of recoloring, which is permitted since the particles are in principle indistinguishable. In the first case shown in Supplementary Movie S2, a recoloring is done whenever two point particles exchange their position numbering along one coordinate axis. For example, if the red-colored particle 9 from the left moves to the right beyond the green particle 10, at the moment when particle 10 becomes the ninth particle from the left it is colored red and particle 9 is colored green. With this recoloring scheme along one coordinate axis, the pseudo-particles defined by the coloring behave as in the one-dimensional case. In the given example, the red pseudo-particle is always number 9 from the left and the green pseudo particle is always number 10 and thus the pseudo-particles are restricted in space by the presence of the others. This argument can be done in both dimensions, and thus the same conclusion as in the one-dimensional case is evident: It is the number of particles that restrict the conformational space of the pseudo-particle of interest, yielding a $N^{N}$ term within the logarithm. Alternatively, the recoloring of the particles is done at each collision between two particles. This means that each particle moves independently of the others as described in Section 2.3 for the one-dimensional ideal gas, but now within a two-dimensional space. Borrowing the derivation from the one-dimensional case (in Section 2.3) with *M* micro-states with $M=\frac{L^{2}}{\Delta x^{2}}$ and *N* collision/recoloring events yielding $\frac{M}{N}$ distinct micro states, we get

(15) $$\Omega ={\left(\frac{M}{N}\right)}^{N}={\left(\frac{L^{2}}{N\Delta x^{2}}\right)}^{N}$$

resulting with Equation (1) in the entropy

(16) $$S^{G}=k_{B}N\left(\ln\frac{L^{2}}{N}+\mathrm{const}.\right)$$

with $N/L^{2}$ the particle density and hence the term in the logarithm is intensive and also shows a $1/N^{N}$ dependency as in the derivations for the one-dimensional gas, yielding again a strictly extensive entropy. It must be noted that for this derivation of the entropy description of a 2D gas, an extension from one to two dimensions has been done entirely by argumentation: By using the Supplementary Movie S2, the independence between the two dimensions is hypothesized, yielding Equation (15), and as such Equation (16) can thus only be regarded a conjecture.

### 2.5. Absence of Ideal Gas Mixing and the GIBBS Paradox in a One-Dimensional System

The so called Gibbs paradox describes the odd finding that by mixing two ideal gases, the Boltzmann entropy will increase only if the two gases are of distinct nature (whatever the distinctness is) and the change of entropy is thereby not dependent on the nature or degree of distinctness [2]. To elaborate on this paradox in the one-dimensional scenario, we consider a box of length L=1 with 2 particles having the same mass *m* with a corresponding box of two particles with unequal mass with $m_{1}>m_{2}$. In Figure 2A, the Sinai billiard representation of the system with equal mass is shown, while in Figure 2B the system with two particles with distinct mass is shown by one particle moving within a right-angled triangle with side lengths $\sqrt{m_{1}}$ and $\sqrt{m_{2}}$. In the latter example after a vertical motion and hitting the hypotenuse, the particle moves in both directions $x_{1}^{\prime }$ and $x_{2}^{\prime }$. This situation translated into the one-dimensional case corresponds to the heavier particle with mass $m_{1}$ coming from the left wall and colliding with the non-moving particle 2 with mass $m_{2}$. After the collision, particle 1 moves back to the left wall, while particle 2 moves to the right wall. The average horizontal and vertical position of the particle is $\langle x_{1}^{\prime }\rangle =\frac{1}{3}\sqrt{m_{1}}$ and $\langle x_{2}^{\prime }\rangle =\frac{2}{3}\sqrt{m_{2}}$, which can either be calculated straightforwardly or by comparing Figure 2A,B using the geometric theorem of intersecting lines. This yields in real space $\langle x_{1}\rangle =\frac{1}{3}$ and $\langle x_{2}\rangle =\frac{2}{3}$ identical to the case of the system with two particles of equal mass (Figure 2A, Equation (7)). Similarly, the standard deviations of particles 1 and 2 are given by $\sigma_{1}^{\prime }=\frac{\sqrt{2}}{6}\sqrt{m_{1}}$ and $\sigma_{2}^{\prime }=\frac{\sqrt{2}}{6}\sqrt{m_{2}}$, which translates to $\sigma_{1}=\sigma_{2}=\frac{\sqrt{2}}{6}$ in the real space, again identical to the result for the system with two equal-mass particles (Equation (14)). It appears obvious that extending this finding to a system of *N* particles with distinct masses represented by a *N*-dimensional simplex with side lengths $\sqrt{m_{p}}$ will still yield Equations (7) and (8) for the mean positions and standard deviations of localization. Hence, the entropy of a mixed one dimensional gas system is the same as its homogeneous analog. Translated to the mixing of two systems, which is the topic of this section, if two one-dimensional gas systems are just mixed, the entropy does not change irrespective of whether the gas molecules are of homogeneous or heterogenous mass.

In summary, in the one-dimensional gas there is not only no Gibbs paradox present, but there exists also no mixing entropy. The latter is due to the impossibility of mixing the positions of the gas particles. This also means that the mixing entropy is solely generated by the mixing of the positions and not by enlarging the accessible volume of each gas upon removing a wall.

### 2.6. Introducing a Mixing in a One-Dimensional Ideal Gas

The situation of mixing two gases in a one-dimensional box discussed in the preceding section demonstrates that a one-dimensional system as described cannot model the mixing happening naturally in a two- or three-dimensional system. In order to do so, we need to introduce a trick to introduce mixing in the one-dimensional gas box. This is possible by letting once in a while two point-particles tunnel freely through each other instead of undergoing a collision between them (as described in Section 2.3 for particles with equal mass). With this concept, the two gases will eventually mix completely irrespective of the frequency or rate and rhythm (periodic, deterministic, or probabilistic) of tunneling. In such a scenario and the principle of thermodynamics to compare only equilibria, the Sinai billiard approach of Figure 2B for two point particles of different mass with the property of both tunneling and colliding needs to be extended to two equivalent triangles facing each other at the hypotenuse as shown in Figure 2C with the collision wall (i.e., the hypotenuse) being absent/porous from time to time. This approach reduces the system not only from two point particles in one dimension to a single one in two dimensions, but directly illustrates the increase of the configurational space of the pseudo-particle by a factor of 2 along with a concomitant increase of the standard deviation of its position as well as the entropy, which changes by a factor of ln2 as expected, because the two triangles are equal (Figure 2C). In the case of two particles with the same mass, the triangle is symmetric in nature and thus the collision wall is not required to be absent from time to time to describe by Sinai billiard a two particle system, yielding no increase of any sort as discussed above. By extension to a 2N-particle system using a 2N-dimensional simplex, it can be demonstrated that this extended one-dimensional gas system can also represent the mixing of two ideal gases with different particle masses. Furthermore, it shows that any mass difference will yield the same entropy increase as expected. The knowledge on distinguishable or not distinguishable particles is required, as either the case of Figure 2A,C needs to be applied, however, without the N! introduced by Gibbs and yielding the factor $N^{N}$ making the entropy again extensive for any number of particles as above.

## 3. Discussion

By studying the entropy of the one-dimensional ideal gas quantitatively and analytically using the Sinai billiard approach, we find that its entropy is extensive for any number of particles *N*. The present derivation shows that the origin of the extensive character of the entropy is due to the $N^{N}$ term within the logarithm and that this is valid for any number of particles. This contrasts with the standard description of the Boltzmann entropy with the initially heuristic introduction of the N! within the logarithm by Gibbs and the rationale for this term to be the indistinguishability of the gas particles.

The one-dimensional ideal gas described here has a volume per particle $V_{p}=V/N$, which is the major determinant of the entropy per particle (via the logarithm). A physical meaning of this entity is, on the one hand, that the number of micro-states is proportional to the volume $V_{p}$ that a single particle is able to access on average, which is inversely proportional to the number of particles *N*, to the power of *N*. This finding also yields zero for the Boltzmann entropy of a fully occupied ideal gas (i.e., every micro-state is occupied by a particle) as one would expect, but which is actually not the case for the Boltzmann entropy calculated by the standard textbook approach (for details see [8]). On the other hand, since the volume $V_{p}$ for particle *p* decreases with increasing number of particles in the system, all the other particles in concert define, by colliding with it, the configurational space of particle *p* as shown in Figure 1. $V_{p}$ can thus be regarded as a measure of the history of the collisions of the particle since the particle is restricted only by its collisions with others. The entropy can therefore be regarded a measure of the potential causal chain of interactions, which is the sum of all the interactions between the particles. This interpretation contrasts with the Boltzmann approach because it relies not on the statistical description of particles moving randomly, with the randomness introduced by collisions, but it compiles and therefore represents a memory of the entire history of a particle’s deterministic trajectory. When time is defined as the metric of causality, the causal history of the system yields an entropy that describes the causal chain between cause and events [19]. While this proposition on the nature of entropy contrasts entirely to the concept of Boltzmann the mathematical formulas are very similar and even the same if in the case of the Boltzmann entropy only the first order term of the Stirling formula is applied, which has its problems though [8]. In this context, it is interesting to note, that the derivation of the Sackur–Tetrode entropy of the ideal gas using quantized space/energy by Sackur used also a volume per particle approach [20]. However, it must also be noted that the one dimensional gas discussed here is only a special case.

The presented calculations also shed light on the so-called Gibbs paradox. At the heart of the Gibbs paradox is the mixing of two gases, which consist of either distinguishable or non-distinguishable particles, yielding in the first case a mixing entropy of 0, while in the second case the mixing entropy is dependent on the volume (and particle numbers of the subsystems). The odd finding is that, based on the standard approach, the mixing entropy is either zero if the particles are strictly indistinguishable or has the same positive value for all the distinguishable cases irrespective of how distinct the gases are. In the first one-dimensional case that we discussed here, this problem does not arise because there is no mixing entropy whether the mass of the two gases are different or not. Only by letting the gas particles tunnel from time to time is a mixing obtained along with a discrete increase of the entropy as calculated in the 3D standard case irrespective on the nature of mixing, but dependent on the particle’s mass difference between the two gases. This finding not only shows that the extended model of the one-dimensional gas appears to recapitulate all the properties of an ideal gas, but also shows that the entropy increase by mixing two gases is due to the mixing per se.

In summary, our study of the one-dimensional ideal gas reveals an extensive entropy that lacks the normalization term N! introduced by Gibbs to resolve the Gibbs paradox. Instead, the normalization factor is $N^{N}$. Of course, the present analysis of a one-dimensional gas is a special case. However, the extended one-dimensional model presented here appears to mimic all essential properties of an ideal gas. Furthermore, thermodynamics must hold for any system since it is “the only physical theory of universal content which […] will never be overthrown, within the framework of applicability of its basic concepts” as stated by Einstein [21], and its constructive microscopic analog—statistical mechanics—should hold for any system covering such complex human enterprises as policy making and sustainability [22], as well as the idealized and rather simple system under study here, the one-dimensional gas. Under the assumption that this special case could be generalized, this would suggest that the Gibbs factor could be in general $N^{N}$ and not N!. While this suggestion contrasts standard statistical thermodynamics (because standard partition functions also contain the N! [3,4,5]), it is in line with another concept of entropy derived by using time as a discrete variable that measures causality yielding an entropy that is a measure of causality [13,14,19] and thus redirects the origin of entropy away from the statistical mechanics approach by Boltzmann towards an argument dependent on the causal history of particles, which is in the example given the collisions between the particles.

## 4. Material and Methods

### A Mathematica Script to Describe Quantitatively the One-Dimensional Ideal Gas

**Figure 3:**
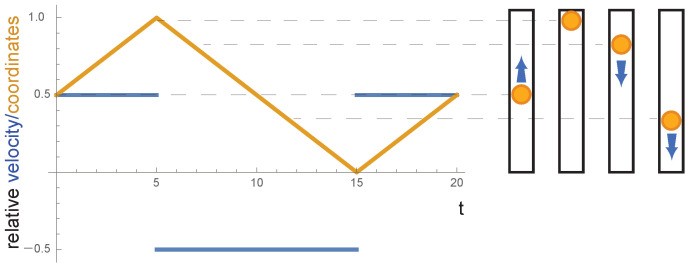
Motion of a single particle in a one-dimensional box of length L=1. The *x* coordinate of the single particle between 0 and L=1 of the box versus time *t* is shown in yellow, while the velocity *v* versus time *t* is shown in blue. In addition, several cartoons of a single particle represented by a yellow sphere within the one-dimensional box show snapshots of the motion of the particle with arrows indicating the direction of the movement. The dashed lines highlight the positions from the cartoons in the graph.

A Mathematica script was established to describe quantitatively a one-dimensional ideal gas comprising *N* particles. As an example, the four-particle system is described here in detail. The velocity of the particle is described by

velocity[initialvelocity_, length_, phaseshift_, time_] :=
initialvelocity SquareWave[(initialvelocity time)/(2 length) + phaseshift]

where initialvelocity is the initial velocity of the particle, length the length of the one-dimensional box, phaseshift the starting position, time the time variable, and the built-in Mathematica function SquareWave that alternates between +1 and −1 with unit period mimics the elastic collisions. Similarly, the position of the particle is described by

position[initialvelocity_, length_, phaseshift_, time_] :=
length TriangleWave[{0,1}, (initialvelocity time)/(2 length) + phaseshift − 0.25]

with the built-in Mathematica function TriangleWave describing the collisions with the wall. Figure 3 illustrates for a single particle the velocity and position over time *t*.

The data in Figure 1B has been obtained from the simulation of a typical case with the following starting conditions of the four particles:

coordinate[20, 10, 0.13, t], coordinate[12.7, 10, 0.41, t],
coordinate[7.31, 10, 0.25, t], coordinate[2.33, 10, 0.05, t]

The positions of the four particles p=1,2,3,4 as a function of time were then evaluated using the expression

RankedMax[{coordinate[20,10,0.13,t], coordinate[12.7,10,0.41,t],
coordinate[7.31,10,0.25,t], coordinate[2.33,10,0.05,t]}, *p*]

This description also integrates collisions between two colored particles as being equivalent to an exchange of the two colors when two (pseudo-)particles pass each other unhindered. In other words, the left-most particle (yellow in Figure 1B) always remains the left-most particle, while the right-most particle (green in Figure 1B) remains always the right-most one, etc.

Next, the time evolution of the system was simulated from time t=0 to 1000 in steps of 0.001 and the probability distributions of the particle positions were evaluated, yielding the histogram with 50 subdivisions along the *x*-axis shown in Figure 1B:

Histogram[Table[RandomVariate[dd[p],10⌃5],{p,1,4}],50]

with

dd[p_] := EmpiricalDistribution[Table[RankedMax[{coordinate[20, 10, 0.13, t], coordinate[12.7, 10, 0.41, t], coordinate[7.31, 10, 0.25, t], coordinate[2.33, 10, 0.05, t]}, p], {t, 0, 1000, 0.001}]]

## Data Availability

Not Applicable.

## Supplementary Materials

The following are available online at: https://www.mdpi.com/article/10.3390/e23091188/s1. Video S1: Movie of the 1D gas highlighting the recoloring concept; Video S2: Movie of a 2D system with the recoloring concept.

## Appendix A

To prove Equation (6), we use Sinai’s result that almost all trajectories of the single particle are ergodic and fill the interior of the *N*-dimensional simplex densely and uniformly. The probability density $f_{p}\left(x\right)$ for the coordinate $x_{p}$ of the particle can therefore be obtained by fixing $x_{p}=x$ while integrating a uniform density over the *N*-dimensional unit simplex:

(A1) $$f_{p}\left(x\right)=N!\int_{0}^{1}dx_{1}\int_{x_{1}}^{1}dx_{2}\cdots \int_{x_{p-1}}^{1}dx_{p}\delta \left(x-x_{p}\right)\int_{x_{p}}^{1}dx_{p+1}\cdots \int_{x_{N-1}}^{1}dx_{N}$$

The normalization factor N! results from the volume 1/N! of the unit simplex. To evaluate this integral, we first consider the *n*-dimensional integral

(A2) $$V_{n}\left(x\right)=\int_{x}^{1}dx_{1}\int_{x_{1}}^{1}dx_{2}\cdots \int_{x_{n-1}}^{1}dx_{n}=\frac{{\left(1-x\right)}^{n}}{n!}$$

as can be seen by induction: $V_{1}\left(x\right)=1-x$ for n=1, and, assuming that the formula is correct for n−1,

(A3) $$V_{n}\left(x\right)=\int_{x}^{1}dx_{1}V_{n-1}\left(x_{1}\right)=\int_{x}^{1}dx_{1}\frac{{\left(1-x\right)}^{n-1}}{(n-1)!}=\frac{1}{(n-1)!}{\left.\frac{{\left(1-x\right)}^{n}}{-n}\right|}_{x}^{1}=\frac{{\left(1-x\right)}^{n}}{n!}$$

Note that $V_{n}\left(0\right)=1/n!$ is the volume of a *n*-dimensional simplex. The integrals over $x_{p+1},\ldots ,x_{N}$ in Equation (A1) can be replaced by $V_{N-p}\left(x_{p}\right)$,

(A4) $$f_{p}\left(x\right)=N!\int_{0}^{1}dx_{1}\cdots \int_{x_{p-1}}^{1}dx_{p}\delta \left(x-x_{p}\right)\;V_{N-p}\left(x_{p}\right)$$

Because of $\delta (x-x_{p})=0$ for $x_{p}>x$, the upper bound 1 of the integral over $x_{p}$ can be replaced by *x*. Since $x_{1}\leq x_{2}\leq \cdots \leq x_{p-1}$, the same applies to the upper bounds of all remaining integrals:

(A5) $$f_{p}\left(x\right)=N!\;V_{N-p}\left(x\right)\int_{0}^{x}dx_{1}\cdots \int_{x_{p-2}}^{x}dx_{p-1}=N!\;V_{N-p}\left(x_{p}\right)\;x^{p-1}\int_{0}^{1}dx_{1}\cdots \int_{x_{p-2}}^{1}dx_{p-1}$$

and hence

(A6) $$f_{p}\left(x\right)=N!\;V_{N-p}\left(x\right)\;x^{p-1}V_{p-1}\left(0\right)=N!\frac{x^{p-1}}{(p-1)!}\frac{{\left(1-x\right)}^{N-p}}{(N-p)!}$$

This completes the proof of Equation (6).
